# Illinois State Medical Society

**Published:** 1889-06

**Authors:** 


### SOCIETY REPORTS.

---

### *ILLINOIS STATE MEDICAL SOCIETY.*

*Thirty-ninth Annual Meeting, Held in Jacksonville, May 21st, 22d and 23d.*

First Day—Morning Session.

The Society was called to order at 10 a.m., by the President, Charles Warrington Earle, M.D., of Chicago.

An address of welcome was delivered by Richard Yates, Esquire.

A response was made by Dr. N. S. Davis, of Chicago.

Dr. T. J. Pitner, Chairman of the Committee of Arrangements, made a number of announcements, after which the President called for the report of the committee on revision of the constitution, which was presented by Dr. T. M. McIlvaine, of Peoria, Chairman.

The report was discussed by Drs. Davis of Chicago, Powell of Collinsville, Herriott of Jacksonville, Ingals, Earle and Graham of Chicago, Darrah of Bloomington, Leeds of Lincoln, Ensign of Rutland, Hunt of Dixon, Carter of Waukegan, Barnes of Bloomington, and Matthews of Carlinville. The matter, on motion of Dr. Graham, was referred back to the same committee, with Drs. N. S. Davis of Chicago and F. B. Haller of Vandalia and J. P. Matthews of Carlinville, as additional members.

First Day—Afternoon Session.

Dr. F. C. Robinson, of Wyanet, Chairman of the Committee on the Practice of Medicine, made his report in abstract which was followed by a paper on

THE TREATMENT OF PNEUMONIA

by Dr. George N. Kreider of Springfield.

Dr. Kreider said that during the past few years much had been written about the mortality of pneumonia. Men whose experience has extended over a long period of years affirm that the death-rate is much higher under the present accepted modes of treatment than it was forty years ago. He advanced the following conclusions:

1. Baths should only be given in those cases which are not progressing favorably.

2. In severe cases nothing is better calculated to give so much relief to all bad symptoms.

3. Inflexible rules for selecting cases and administering baths cannot be given. The thermometer is not always a guide for their administration. Difficulty of respiration and lack of secretion should lead to their employment regardless of the height of the temperature.

4. The bath should be given at a temperature of 100° F., or less, depending on the severity of the case and the condition of the patient. He had not found baths of extremely low temperatures necessary as yet, but should not hesitate to employ them if occasion should require.

5. Stimulants should be given just before entering and in coming out of the bath-tub. The patient should not make any exertion himself.

6. The physician should himself superintend and aid in giving the bath. Exception to this rule should only be made when the patient is under the care of trained nurses who are competent to meet emergencies which may arise.

7. The bath treatment is capable of shortening the duration of the disease and of convalescence, and of reducing the mortality. Liebermeister says mortality has been reduced in his hands from twenty-five to ten per centum. The ice coil will be found effective in the milder cases in reducing temperature and relieving pain, if placed directly on the affected side.

Dr. J. M. G. Carter, of Waukegan, said that anything which will tend to equalize the circulation in pneumonia will be beneficial. Whenever a hot or a cold bath, or local application, will accomplish that purpose, it is a good remedy.

In regard to diphtheria, he had been unable to determine its contagiousness. In the treatment he used aconite to reduce temperature; belladonna to control the condition of the throat and to act as an anodyne, because most sore throats occurred in children. He gives them chlorate of potash mixed with sugar. He does not recommend gargles, except where they can be used easily.

Dr. J. S. Miller, of Peoria, had seen but two well-defined cases of diphtheria during the last three or four years. He would refer to one as "masked" diphtheria, because he treated the case three or four days before he was able to make a diagnosis, and it was only a few hours before the death of the patient that he was able to find out the true condition. The symptoms were those of fever and difficulty in breathing. He was unable to detect any lung trouble. A few hours before death of the child he discovered diphtheritic membrane clinging to the throat. A great deal of attention should be paid to diagnosis in such cases. Relative to treatment, he was of the opinion that time is one of the most important factors. If we can gain time we do more toward guiding the case to a successful issue than by medication, and in order to gain time he favors the supporting plan of treatment. He does not believe in specifics in diphtheria. Iron, quinine, and whisky should be given to prevent the destruction of tissue. One of the valuable local applications is ice. He has discontinued local applications to the throat entirely.

Dr. W. M. Barritt, of Onarga, in the treatment of diphtheria, impresses the system as early as possible with some antiseptic, preferably the biniodide of mercury, it being more diffusible than some other forms. In early childhood where it is not possible to make application locally, he depends mainly upon constitutional treatment. Sulphurous acid, administered internally, gives good results.

Dr. J. Townsend, of Springfield, said that tepid applications were very beneficial in reducing the temperature in pneumonia. He had seen numerous instances in which the use of the coil in cases of puerperal fever and typhoid fever proved very beneficial.

Dr. W. T. Thackeray, of Chicago, said that antipyrin reduces temperature rapidly, but the ill effects upon the stomach and the depressing influence upon the nervous system lead physicians to question its value as an antipyretic. He had never known it to maintain an excessively low temperature, say 96 or 97. He had known the late Dr. Byrd, of Quincy, to use the cold pack with excellent results.

Dr. David Prince, of Jacksonville, referred to the question of temperature in disease, and said the cold coil is applied to a patient with inflammation of the brain, and relief is afforded; a hot coil is applied, and there is relief. It is evidently not the amount of caloric heat abstracted from the body, but some local influence which is transmitted to some other center which results in the contraction of the vessels in both instances, where a hot or cold coil has been used.

With reference to the treatment of diphtheria, the best success he had ever seen was by the use of a spray of Lugol's solution. He had seen some remarkable recoveries from this spray. In an epidemic he had once used bromine in the same way, supposing it to be a better antiseptic than iodine, but his practice did not bear out the anticipation.

Dr. A. J. Baxter, of Astoria, is in the habit of giving warm baths in pneumonia; also in cases of typhoid fever, alternated with cold, and he finds almost as much benefit from one as the other.

In the use of antifebrin and antipyrin, much depends upon the condition of the system in which they are used. In very nervous patients he is more cautious in administering them than in those of a robust constitution.

Dr. L. L. McArthur, of Chicago, said that the variety of abscess of the lungs which results from pneumonia is simply of a suppurative character, non-tubercular, and too frequently a diagnosis is not made. In addition to the treatment recommended by Dr. Robinson, it is now considered good practice to open such abscesses of the lungs and drain them as in any other portion of the body.

The papers were further discussed by Drs. E. P. Cook of Mendota, A. F. Lee of Quincy, and S. T. Hurst of Greenview.

Dr. A. J. Baxter, of Astoria, read a paper on

DYSPEPSIA.

Dr. N. S. Davis, of Chicago, said he knew of a great many conditions of the digestive organs that go under the general designation of dyspepsia; that the only successful method in treating such cases is to analyze each case on its merits, to find out the habits and pathological condition of the digestive organs and rationally adjust the treatment accordingly.

The paper was further discussed by Dr. A. Wetmore of Waterloo.

Dr. J. L. White, of Bloomington, read a paper entitled

HYGIENE AND DOCTORS' FEES.

which was discussed by Drs. Kreider, Davis, and G. L. Eyster.

The President's address on

THE DUTIES AND RESPONSIBILITIES OF THE MEDICAL PROFESSION REGARDING ALCOHOLIC AND OPIUM INEBRIATION, was delivered during the evening session. He claimed that alcoholism is not a disease. Ninety-nine out of a hundred drunkards show no symptoms of disease. A man can cure himself of the habit by exercise of the will unaided by medicine, and no disease can be cured that way. No physical cause compels a man to become a slave. Ninety-nine out of a hundred men can reform if they will. It requires a cultivation of the moral sense, a strengthening of the will, a cutting off of all evil associations and all bad habits and total abstinence from the use of liquor. A man should carefully avoid all temptation. It will not do for him to attempt to drink in moderation. It is true that alcoholism may produce changes in every tissue, and bring about all manner of diseases, but it is not a disease itself, and a man who has drank ten, twenty or thirty years may stop as easily as a man who has drank only three months. Dr. Earle claimed that neither the alcoholic nor morphine habit is hereditary, and he quoted figures to prove it. Idleness and want of government are, he said, more prolific causes of drunkenness. The most marked results of inebriety are not physical, but mental and moral. They are not productive of disease to the extent we are led to believe. Physicians should be temperate for their own sakes, and also to set examples for others. They should be more careful how they prescribe alcohol and morphine. The last is the more seductive, and physicians are responsible for the morphine habit in a majority of cases. It should only be prescribed in perilous cases, and not at all for trivial complaints, as is too common. He advised that the young be educated to avoid the habits; that work be prosecuted among those who earnestly desire reformation, and that legislation be sought for the incorrigible class who cannot be reformed, and for them he recommended state guardianship, and that they be placed for two years in an institution located on a farm.

### Second Day—Morning Session.

The report of the Committee on Diseases of Children was made by Dr. A. T. Darrah, of Bloomington.

Under the general term of *summer complaint*, are grouped such diseases as diarrhœa, dysentery, cholera infantum, and entero-colitis. Of the great mortality of children under the age of five years vital statistics show that a large percentage is caused by bowel diseases during the hot weather. This is especially marked in the larger cities of the central and southern parts of the United States.

Heat weakens by destroying the cohesive force between atoms. The result is a relaxed and flabby condition of the tissues. Continuous high temperature, acting upon the very delicate nervous organization of children has the effect of destroying the digestive ferment of the stomach, or of arresting its natural secretion, and then fermentation may take its place. Heat being the prime factor in the causation, and acting through the sympathetic nervous system, its effects should be counteracted, hence *cold* is the remedy. By reducing the temperature the cause of the disease is removed. We should not wait during warm weather for the little ones to become sick, but prevent it by beginning the use of baths with tepid, or cool or cold water, using it freely, and letting the temperature be suited to each individual case.

Dr. A. E. Hoadley, of Chicago, said that after an experence of twelve years in treating diarrhœa in infants, he could testify to the utility of the treatment advocated by Dr. Darrah. In his practice he teaches families to keep the babies cool. As soon as they begin to feel the effects of heat, their faces red and their head tossing about, crying, etc., instead of giving them extra food of some kind, part of their clothing is removed. If the weather is excessively hot they take off more clothing. The children are then sponged with tepid water, and allowed to lie on the floor naked, during the great heat of the day. Towards evening, when the weather becomes cooler, clothing is put on them. With such treatment the children had little or no diarrhœa, or indigestion, or like trouble during the summer. He is rarely called to see a case of acute diarrhœa in infants during the summer.

Dr. Robinson, of Wyanet, called attention to the fact that many parents refused children water to drink when they have diarrhœa. He advocates giving them all the water they can possibly drink.

Dr. Baxter and Dr. Davis also took part in the discussion.

Dr. W. T. Thackeray, of Chicago, presented a report as Chairman of the Committee on Drugs and Medicines.

He said among the most important introductions during the year is that of Professor Pribram, of Berlin—viz., *agaricine*, an alkaloid from white agaric, touch-wood, tinder from the torch tree. This fungus was orginally employed by Andral as a remedy against night-sweats, and has attained some reputation in this direction in Europe. In America, however, the agaric was only used to a limited extent as a purge. It was early discovered that two distinct resins were contained in the fungus, one practically insoluble, and the other soluble in alcohol and chloroform, the product being a yellow crystalline powder and containing what medicinal virtue was possessed by the drug. From this principle Pribram isolated the alkaloid agaricine, and he epitomized his experience with it as follows: It always decreases the sweat; thirst and the excretion of urine are diminished under its use; the functions of the lungs and skin are not interfered with; it gives no after ill-effects.

Dr. Thackeray had found it efficacious in the dose of two milligrammes, in several cases. Others reported, however, that they had used as much as eight milligrammes before the effects were produced.

A paper entitled

NOTES ON THE CAUSES AND MANAGEMENT OF HERNIA IN INFANTS AND YOUNG CHILDREN, by Dr. K. Miller, of Lincoln, was read by Dr. Leeds in the absence of the author.

A paper on

VAGINAL HYSTERECTOMY, by H. T. Byford, of Chicago, was read by Dr. F. H. Martin, in the former's absence.

The death rate of recent vaginal hysterectomies is about ten per centum, while that of some operators is even lower and steadily decreasing. The steps of the operation are as follows:

1. An incision is made through the vaginal wall extending in a circular direction two-thirds the distance around the cervix in front and at the sides.

2. Separation of the parametric tissue from the uterus up to the uterine arteries at the side, and the bladder from the uterus in front until the peritoneum is reached.

3. Completion of the circular incision behind the cervix.

4. Opening the peritoneal cavity posteriorly and anteriorly.

5. Ligation or clamping of broad ligaments, and severing them from the uterus.

6. Tamponment of the vagina with iodoform gauze.

The speaker would favor giving vaginal hysterectomy a trial in all cases of uterine cancer, and particularly in cases discovered early. In view of the diminishing death rate he would perform the same operation for small fibroids, which resisted treatment and showed a tendency to rapid growth, or produce incurable distressing symptoms. Occasionally cases of adenoma uteri, metrorrhagia, displacements, etc., may require the operation, not to cure a fatal disease, but to relieve a life of suffering and invalidism otherwise unavoidable.

Dr. C. Truesdale, of Rock Island, read a paper on the

ETIOLOGY AND PATHOLOGY OF UTERINE DISPLACEMENTS.

He summarized his remarks as follows:

1. The normal typical position of the uterus in the pelvis in an erect position of the body, instead of being upright, is nearly horizontal.

2. So-called anteversions of the uterus pathologically have no existence.

3. The body of the uterus is supported by the bladder, the bladder upon the vagina, the vagina upon the perineal septum as far back as the rectum, the neck suspended by the utero-sacral ligaments on a plane as high as the body with the empty bladder.

4. All ordinary displacements, such as prolapsus, anteflexion, retroversion, and retroflexion, are produced by contracture of the muscular walls of the vagina.

Dr. Franklin H. Martin, of Chicago, read a paper in which he reported three cases of fibroid tumors of the uterus operated upon by Apostoli's method, which illustrated the three different procedures of the method, viz:

1. Intra-uterine galvano-caustic—positive operation.

2. Intra-uterine-galvano-caustic—negative operation; and

3. The intra-vaginal galvano-puncture operation.

He also gave a short summary of results obtained by himself in a series of one hundred consecutive cases, treated by Apostoli's method which were as follows:

1. Of the hæmorrhagic cases, 95 per centum were cured of hæmorrhage.

2. Tumors entirely disappeared in 80 per centum.

3. Tumors were reduced in size in 78 per centum.

4. Tumors not affected in size either from failure of treatment, or from patients discontinuing treatment, or for other reasons, 14 per centum.

5. Neuralgic and other pains relieved in 90 per centum.

6. No deaths.

7. Symptomatic cures, 68 per centum.

Dr. L. L. McArthur, of Chicago, read a paper on

UTERO-VAGINAL FISTULA AND ITS TREATMENT, WITH REPORT OF A CASE.

He emphasized some points in the technique of the operation, as follows:

1. Suturing should be in the longitudinal rather than in the transverse diameter of the vagina, because there is less contraction on the stitches and greater facility in introducing them.

2. Choice of material for sutures varies, though silk-worm gut has perhaps the preference, Sims, Bozeman, and Haegar preferring silver wire. Pippingsjold claims excellent results from the use of alternate silver and iron wire, believing that a galvanic current is thus induced which plays an important part in the union. Excellent results are on record with silk as the suture.

3. Simon, perhaps, did more than any other operator, to simplify the treatment. He demonstrated that it was neither necessary to retain a catheter in the bladder after operation, nor to maintain the reclining posture. He permits his patients to rise as soon as they feel disposed, simply requiring them to empty the bladder at least every two hours.

4. It is advantageous to render the urine antiseptic by some such agent as saccharine, benzoic acid, or boracic acid internally.

5. If the first operation succeeds in locating the orifice of the fistula on the mucous surface of the bladder, that constitutes one step in some methods; then, at a later sitting, closing the bladder-opening may be effected.

6. Any colored liquid may be used to differentiate the fistula, as those above mentioned.

7. It is not necessary to put off the cure until the age of puberty is reached.

8. The orifice of the fistula should be encircled with about one-quarter inch of mucous membrane. This serves to retain the ureter in the bladder and prevents, by a valve-like action, the escape of urine through the wound.

9. It is to be inferred that the sphincter vesicæ, in a congenital case, has never been trained to its proper function and may need attention in the line of internal medication.

Second Day—Afternoon Session.

Dr. O. B. Will, of Peoria, chairman, presented the report of the Committee on Obstetrics.

He said with respect to the use of anæsthetics, ergot, and the forceps, in facilitating labor and lessening suffering, there has always been, at least, some diversity of opinion. Where, however, the pains are severe it is not now considered necessary to permit a woman to suffer and become nervous and excited over the so-called "nagging" pains of the first stage of labor, any more than those of later and severer type. On the contrary, it is not considered a mere matter of choice, but of justice and necessity. Until comparatively recent years, chloroform has generally been considered the best anæsthetic. In the estimation of the writer, the so-called *A. C. E. mixture* of alcohol, chloroform, and ether in the ratio of one, two, and three parts, seems to leave little to be desired. The next popular agent is hydrate of chloral. Its use is preferred by many to chloroform during the last expulsive pains of labor. "Wisdom, says Dr. Peak, "should teach us that chloroform should be used in all cases of labor where not contra-indicated by,

"1. Organic diseases in the brain ;

"2. Destructive lesions in the lungs ;

"3. Fatty degeneration of the heart ;

"4. Extreme general anæmia."

The report closed with the following suggestions :

(*a*) When the proper time arrives the head should be kept in constant contact with the perineum in the intervals of pain, with the hand or the forceps, as the case may be.

(*b*) The condition of the parts should be always known.

(*c*) Whenever possible, the head should pass the outlet during the intervals between pains.

Dr. A. F. Rooney, of Quincy, contributed a paper entitled

THE USE OF THE CURETTE IN PUERPERAL ENDOMETRITIS.

Whenever there is fever or pain or fetor which points to this cause, the quickest and the surest way to abort the trouble is to carefully remove, with a dull curette, every easily-detachable fragment. There need be no violence, and there should be no fear of consequences.

Dr. Rooney finds it more convenient to take advantage of Sim's position to steady the cervix with a tenaculum, to bend the curette to that curve which allows it to pass the os internum most readily and find the irregular surfaces, and by this means roll out any tissue-fragments, or dark clots that may be enclosed, for sometimes the cervix contracts down quite firmly, and the uterus, especially in miscarriages, appears flexed either forward or backward. After curetting, Dr. Rooney applies iodized phenol upon a cotton wrapped applicator, regarding it as a valuable antiseptic and styptic. A Vienna physician had reported two hundred cases in which he had curetted for puerperal endometritis with no deaths.

Dr. C. C. Hunt, of Dixon, read a paper entitled

CARBOLIC ACID, PURE OR IN CONCENTRATED SOLUTION, AS A DISINFECTANT IN THE TREATMENT OF PUERPERAL SEPTICÆMIA, in which he said the following cardinal points are now generally admitted :

1. Puerperal septicæmia is of bacterial origin.

2. It is identical with surgical fever, but under exaggerated conditions.

3. It is preëminently infectious.

4. It is preventable.

During the spring of 1888 he had seen a great many interesting cases.

His clinical experience with undiluted or very strong solutions of carbolic acid tended to establish :

1. That it is not attended with danger.

2. That if applied early the temperature is immediately reduced.

3. That it is efficient as a disinfectant; no micro-organisms or spores can exist or develop where it touches.

4. That to apply it effectually and safely we must be guided by the sense of sight as well as that of touch.

SECOND DAY—EVENING SESSION.

Dr. R. H. Engert, of Chicago, read a paper on the

RELATION OF UTERINE FIBRO-MYOMA TO THE ORGANIZATION OF THROMBI, in which was advanced the opinion that a fibroid is nothing more or less than an organized giant thrombus.

To substantiate this hypothesis, quotations were made from some of the latest researches in regard to the organization of thrombi. Welch, of Baltimore, writes: "In a thrombus of five minutes existence, blood corpuscles were found in a state of degeneration. Leucocytes were already visible, although sparingly. Half an hour later they increased considerably in number. Fibrin appeared in from five to thirty minutes in the form of strips and islets between the blood corpuscles and on the surface of the blood coagula. Gradually it formed a net-work, and twenty-four hours from the beginning of the process a great part of the thrombus consisted of fibrin. Leucocytes seemed to wander about in the coagula and the fibrin seemed to increase in proportion to the increase of these."

Heuknig holds that the "first process in the organization of a thrombus is the conversion of the blood into a fine granular mass, this being hyaline and striated. Canalization takes place from the line of demarkation on the border of the vessel wall, proliferation of the endothelium begins, and gradually covers that part of the thrombus which does not adhere to the surrounding wall. Underneath this cover a layer of connective tissue develops. The endothelium begins to grow into the thrombus and capillaries are formed. Another set of capillaries develops from the vasa vasorum of the vessel in that particular thrombus which adheres to the vessel-wall. Gradually the connective tissue increases while the thrombus decreases."

Dr. Engert then reported some cases tending to substantiate the previously advanced statements.

Dr. N. S. Davis presented a report on "Meteorological Conditions and their Relation to the Prevalence of Acute Diseases."

Dr. D. R. Brower, of Chicago, followed with some remarks on the subject of "Writer's Cramp," and exhibited an appliance devised by Nussbaum.

Third Day—Morning Session.

Dr. Edmund Andrews, of Chicago, made a report of the results of

ONE HUNDRED OPERATIONS FOR STONE IN THE BLADDER.

He said the object of the paper is to compare the safety of litholopaxy with that of lithotripsy and of lithotomy in his own practice. He had operated for stone over one hundred times, employing all the different methods in vogue.

The following is a summary of results: Litholopaxy (Bigelow's Operation), forty cases, one death; mortality, two and a half per centum. Lithotrity (after Sir Henry Thompson's method), six cases, one death; mortality, seventeen per centum. Lithotomy below age of puberty, fifty-five cases, seven deaths; mortality, thirteen per centum. Lithotomy above age of puberty, twenty-nine cases, five deaths; mortality, seventeen per centum.

His experience in the old form of lithotrity of one death in six operations is substantially like that of other surgeoms. Litholopaxy can generally be performed only in adults. Statistics of adult lithotomy over the whole globe, amounting to some four thousand cases, indicate an average mortality of from twelve to eighteen per centum, while litholopaxy in Chicago—at least in his practice—forty cases had a death-rate of only two and one-half per centum. The most remarkable successes claimed for lithotomy come from the warmer regions of Asia, where calculi seem to be very abundant.

The following are gathered from authentic sources: Baboo Ram Norain Das Bahadore, of India, cases, 248; deaths 17; mortality, 7 per centum. Dr. Kerr, American Hospital, China, cases, 187: deaths, 19; mortality, 10 per centum. Dr. Nishman Altoungan, Amasia, Asia Minor (educated at the American Mission), cases, 265; deaths,

5; mortality, 2 per centum. Dr. Sewny, of Livas, Asia Minor, cases, 40; deaths, 1; mortality, 2½ per centum. Total number of cases, 740; deaths, 42; mortality, 6 per centum.

Dr. A. E. Hoadley, of Chicago, read a paper on

TUBERCULAR JOINT-DISEASE.

He said this is by far the most common joint-affection in early life. Tuberculous inflammations of joints are characterized by the rapidity with which they run their course. Three or four weeks is sufficient time for tubercular inflammation, if complicated with staphylococcus, to completely destroy all the elements of a joint, synovial membrane, ligaments, cartilage and bones, rendering the entire joint a structureless mass which communicates with the outside by several openings. The disease may have its starting point in the epiphyseal cartilage or in the synovial membrane, and in the bones themselves, but never in the articular cartilage or the articular ligaments. Both the latter are readily destroyed by infection. In seventy-seven per centum of all cases, whether rapid or slow, the affection remains local, in the remainder the tuberculosis becomes general.

He emphasized the fact that early, thorough, and repeated aseptic operations is the treatment for this class of cases. There is at the present time but one fixed rule in regard to the kind of operation to be performed, and that is to remove all the diseased tissue in whatever structure it may exist and sacrifice nothing,—that is, remove any healthy tissue, bone especially, that is unquestionably in the way and liable by its presence to interfere with obtaining the best results. Excision of joints in these cases, as a rule, is objectionable, because of the unnecessary amount of healthy bone removed; complete enucleation of the synovial membrane together with such diseased portions of bone as may exist gives the best results. The operation should be repeated as often as it becomes evident that the disease is still in progress.

Dr. H. M. Starkey, of Chicago, Chairman, made the report of the Committee on Ophthalmology and Otology.

He directed attention to the advisability of having the eyes examined in every case of persistent headache not relieved by the ordinary medical means, even when the eyes are not obviously at fault.

Dr. Galezowski called attention to the close relation between some eye-troubles and irritation of the upper teeth.

The only new drug that seems to have met with any special favor is the antiseptic creolin.

Dr. Isadore Berrman recommends the use of lactic acid in from ten to thirty per centum solutions for cases of chronic suppurative otitis that have resisted other treatment.

Dr. Lyman Ware, of Chicago, read a paper on

SYPHILITIC CYCLITIS, and reported several cases. He offered the following suggestions:

1. Greater care in the diagnosis of primary symptoms. However difficult it may be to diagnosticate primary or secondary syphilis, it is generally far more difficult to diagnosticate tertiary. It is much too common for a physician to say to his patient: "I do not think you have syphilis, but yet a little constitutional treatment will do no harm."

2. Mercury is unquestionably best in the primary and secondary stages, while potash in large doses is the remedy in the tertiary stage.

3. Large doses of potash can only be tolerated when given in gradually increasing doses, preferably after meals, in much water; the freer the perspiration, the larger the doses of potash.

OFFICERS ELECTED FOR THE ENSUING YEAR ARE:

*President*—Dr. John Wright, of Clinton.

*First-Vice-President*—Dr. J. P. Mathews, of Collinsville.

*Second-Vice-President*—Dr. T. M. Cullimore, of Jacksonville.

*Permanent Secretary*—Dr. D. W. Graham, of Chicago.

*Assistant Secretary*—Dr. L. Ware, of Chicago.

*Treasurer*—Dr. T. M. McIlvaine, of Peoria.

The next meeting of the Society will be in Chicago on the third Tuesday in May, 1890.

